# Independent Clinical Validation of the Automated Ki67 Scoring Guideline from the International Ki67 in Breast Cancer Working Group

**DOI:** 10.3390/biom11111612

**Published:** 2021-10-30

**Authors:** Ceren Boyaci, Wenwen Sun, Stephanie Robertson, Balazs Acs, Johan Hartman

**Affiliations:** 1 Department of Clinical Pathology and Cancer Diagnostics, Karolinska University Hospital, 11883 Stockholm, Sweden; ceren.boyaci@ki.se (C.B.); wenwen.sun@ki.se (W.S.); stephanie.robertson@ki.se (S.R.); 2 Department of Oncology and Pathology, Karolinska Institute, 17177 Stockholm, Sweden; 3 Medtech Lab, Bioclinicum, Karolinska University Hospital, 17164 Stockholm, Sweden

**Keywords:** Ki67, breast cancer, QuPath, digital image analysis, validation, reproducibility

## Abstract

Ki67 is an important biomarker with prognostic and potential predictive value in breast cancer. However, the lack of standardization hinders its clinical applicability. In this study, we aimed to investigate the reproducibility among pathologists following the guidelines of the International Ki67 in Breast Cancer Working Group (IKWG) for Ki67 scoring and to evaluate the prognostic potential of this platform in an independent cohort. Four algorithms were independently built by four pathologists based on our study cohort using an open-source digital image analysis (DIA) platform (QuPath) following the detailed guideline of the IKWG. The algorithms were applied on an ER+ breast cancer study cohort of 157 patients with 15 years of follow-up. The reference Ki67 score was obtained by a DIA algorithm trained on a subset of the study cohort. Intraclass correlation coefficient (ICC) was used to measure reproducibility. High interobserver reliability was reached with an ICC of 0.938 (CI: 0.920–0.952) among the algorithms and the reference standard. Comparing each machine-read score against relapse-free survival, the hazard ratios were similar (2.593–4.165) and showed independent prognostic potential (*p* ≤ 0.018, for all comparisons). In conclusion, we demonstrate high reproducibility and independent prognostic potential using the IKWG DIA instructions to score Ki67 in breast cancer. A prospective study is needed to assess the clinical utility of the IKWG DIA Ki67 instructions.

## 1. Introduction

Ki67 is a non-histone protein that plays an important role both in cell division and during interphase, while its localization in the nucleus changes constantly [1]. In pathology practice, Ki67 is often used to evaluate cell proliferation by assessment of protein expression in actively dividing cells based on immunohistochemistry, which is an easily accessible technique. Ki67 is scored by calculating the percentage of positively stained tumor cells, generally referred to as the “Ki67 proliferation index”.

The immunohistochemical determination of Ki67 gained increased attention after the proposal from the St. Gallen consensus guideline statement in 2011, where Ki67 was recommended to be used for dividing breast cancers into “surrogate intrinsic subtypes” for therapeutic purposes [2]. The usage of Ki67 in breast cancer management has thereafter been controversial but holds a promising role in the prediction of chemotherapy response [3].

However, the standardization of pre-analytical processes and the interpretation of Ki67 scoring have been intensely discussed [4]. There is no widely applied consensus as to whether the whole tumor area or hotspots should be evaluated [4]. Moreover, manual assessment in hotspot areas is the most commonly used method but is subjective in nature reflecting its person-dependent design [5]. The need for a standardized assessment method is of utter importance.

In the search for a gold standard for reliable Ki67 scoring, digital image analysis (DIA) platforms provide several opportunities. DIA platforms have been shown to increase reproducibility between observers and to improve intra-observer correlations [6,7]. Yet these machine-read methods require clinical validation.

Recently, a guideline for setting up an open-source automated Ki67 scoring algorithm was introduced by the International Ki67 in Breast Cancer Working Group (IKWG), and an analytical validation study was performed with high inter-laboratory reproducibility [8]. In the present study, we aimed to investigate the reproducibility among pathologists following this image analysis guideline for Ki67 scoring and to evaluate the prognostic potential of the suggested platform in an independent cohort.

## 2. Materials and Methods

### 2.1. Patient Cohort

The study comprises a previously published cohort including a total of 222 patients diagnosed with invasive breast carcinoma at the Karolinska University Laboratory, Sweden, from 2002 to 2009 and the Stockholm South General Hospital, Sweden, in 2012 [9,10,11,12,13]. From this cohort, a total of 157 tumors were available for DIA after the cases with poor immunohistochemistry quality, without any invasive carcinoma on the slide and cases of HER2+ and triple-negative subtypes were excluded. Clinicopathological data included up to 15 years of follow-up outcome data was obtained from the pathology laboratory information system and the medical record system

### 2.2. Immunohistochemical Staining

Tissue serial sections were retrieved from formalin-fixed paraffin-embedded tumors at the clinical laboratory of the Department of Pathology, Karolinska University Hospital, Sweden. The sections were stained with a rabbit monoclonal anti-Ki67 antibody, clone 30-9 (Ventana Medical Systems, Tucson, AZ, USA) within the routine breast cancer panel according to the manufacturer’s protocol as previously described [10]. The cut-off value for Ki67 was defined as 20% (<20% for Ki67 low group and ≥20% for Ki67 high group) [14].

### 2.3. Digital Image Analysis

**Figure 1:**
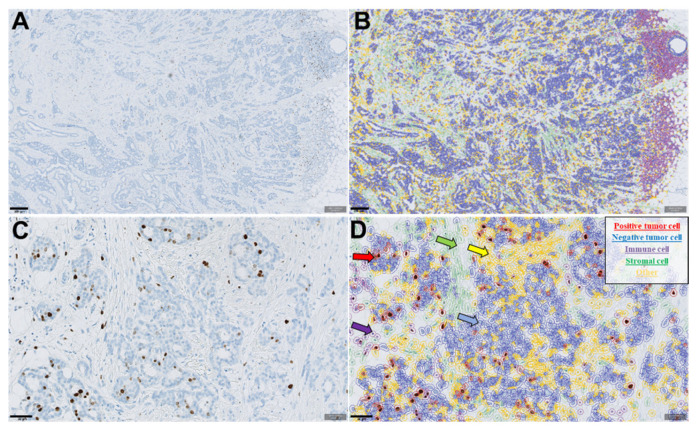
(**A**,**C**) Digitized images of Ki67 immunohistochemistry-stained breast tumor used in the study. (**B**,**D**) Corresponding images of (**A**,**C**) in QuPath after cell segmentation and classification. Different colors represent different cell types: red color shows Ki67-positive tumor cells, blue shows negative tumor cells, green indicates stromal cells, purple marks lymphocytes and yellow represents background/false detections.

The Hamamatsu platform (Hamamatsu Photonics, Japan) was used at ×40 to digitize the histological slides [Ki67, and hematoxylin and eosin (HE)] with a pixel size of 0.4986 × 0.4986 µm. The QuPath DIA platform was utilized to score average tumoral Ki67 expression using the guideline from the IKWG (https://www.ki67inbreastcancerwg.org/) (accessed on 30 August 2021). Briefly, after the whole invasive cancer area was annotated, the “estimate stain vectors” command was used to refine the hematoxylin and DAB stain estimates for each case. Watershed cell detection [15] was used to segment the cells in the digitized slide with the following settings: detection image, optical density sum; requested pixel size, 0.5 µm; background radius, 8 µm; median filter radius, 0 µm; sigma, 1.5 µm; minimum cell area, 10 µm^2^; maximum cell area, 400 µm^2^; threshold, 0.1; maximum background intensity, 2. To classify the detected cells into tumor cells, immune cells, stromal cells and others (background/false detections), we used random trees as a supervised machine learning method. The features used in the classification are shown in Supplementary File 1. Following the guideline, one breast cancer case with a whole-slide section (WS) was selected independently from the study cohort for algorithm training (Figure 1). Thereafter, a total of four algorithms were created and independently trained by two board-certified breast pathologists and two resident pathologists, one of whom has a PhD in digital image analysis (a total of four pathologists). These DIA Ki67 scoring algorithms were locked down and applied to the study cohort. The reference Ki67 scores of the study cohort were obtained by a separate QuPath algorithm independently trained only on the study cohort (30 randomly selected cases) and were used for comparisons.

### 2.4. Statistical Analysis

The reproducibility among pathologists was estimated by calculating an ICC (intraclass correlation coefficient). We considered ICC values between 0.4 and 0.6 as having moderate reliability, values between 0.61 and 0.8 as having good reliability and values greater than 0.8 as having excellent reliability [16]. Pre-specified criteria of success were defined as ICC on log-transformed Ki67 values with a lower limit of 95% confidence interval (CI) ≥ 0.80. Kaplan–Meier analysis supported with log-rank test was executed to assess prognostic potential. The Cox proportional hazard model was used to test independent prognostic potential. Relapse-free survival (RFS) was defined as time from the date of primary diagnosis to the occurrence of first relapse.

In all statistical analyses, the level of significance was set at *p* < 0.05. All statistical analyses were performed in SPSS 22 software (IBM, Armonk, NY, USA).

## 3. Results

### 3.1. Patient and Tumor Characteristics

In the study cohort of 157 cases, the mean age of patients at diagnosis was 59 years and the median follow-up time was 8.84 years. The mean tumor diameter was 25 mm, and the median tumor diameter was 22 mm. Twenty-seven tumors were histological grade 1, 84 tumors were grade 2 and 46 tumors were grade 3 according to the Nottingham histological score. The pathological tumor-node-metastasis (pTNM) classification based on the eighth edition of the American Joint Committee on Cancer (AJCC) breast cancer staging system showed that 63 cases were pT1, 86 cases were pT2 and 8 cases were pT3. Furthermore, 87 cases had no metastasis, 51 cases had 1–3 lymph node metastases, 15 cases had 4–9 lymph node metastases and 4 cases had 10 or more lymph node metastases (Table 1).

### 3.2. Reproducibility among Pathologists

**Figure 2:**
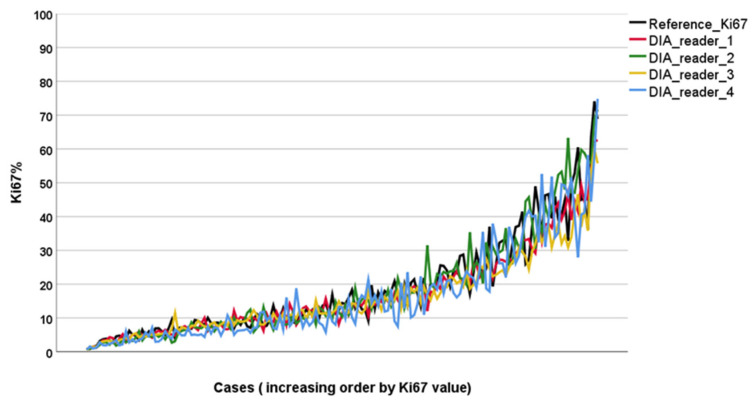
The distribution and variability of Ki67 scores. Each line represents Ki67 scores from one algorithm/pathologist. Cases are ordered by increasing Ki67 value.

**Figure 3:**
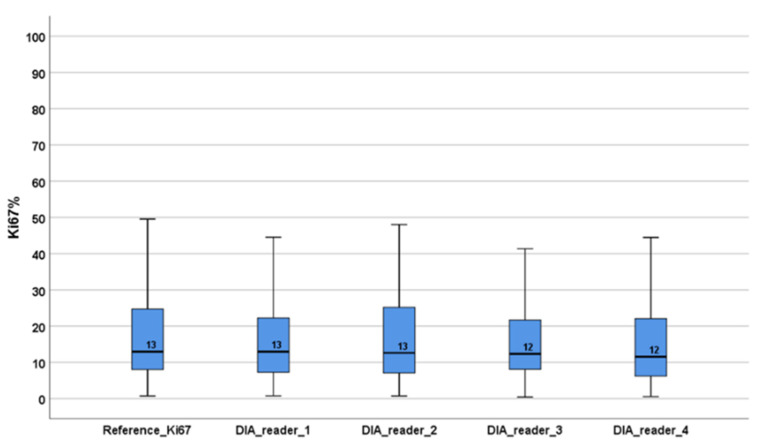
The distribution of Ki67 scores for the respective algorithms (reference and pathologists 1–4).

High interobserver reliability was found with an ICC of 0.938 (CI: 0.920–0.952) among the reference standard score and the four Ki67 algorithms built following the detailed guideline from the IKWG (Figure 2). The distributions of the Ki67 scores across the four algorithms and the reference score were similar. The median Ki67 values ranged between 12 and 13% (Figure 3). The median tumor cell count with DIA global scoring in QuPath was 122,465 cells (range: 2346–996,783 cells).

### 3.3. Prognostic Potential of DIA Ki67 Scoring

**Figure 4:**
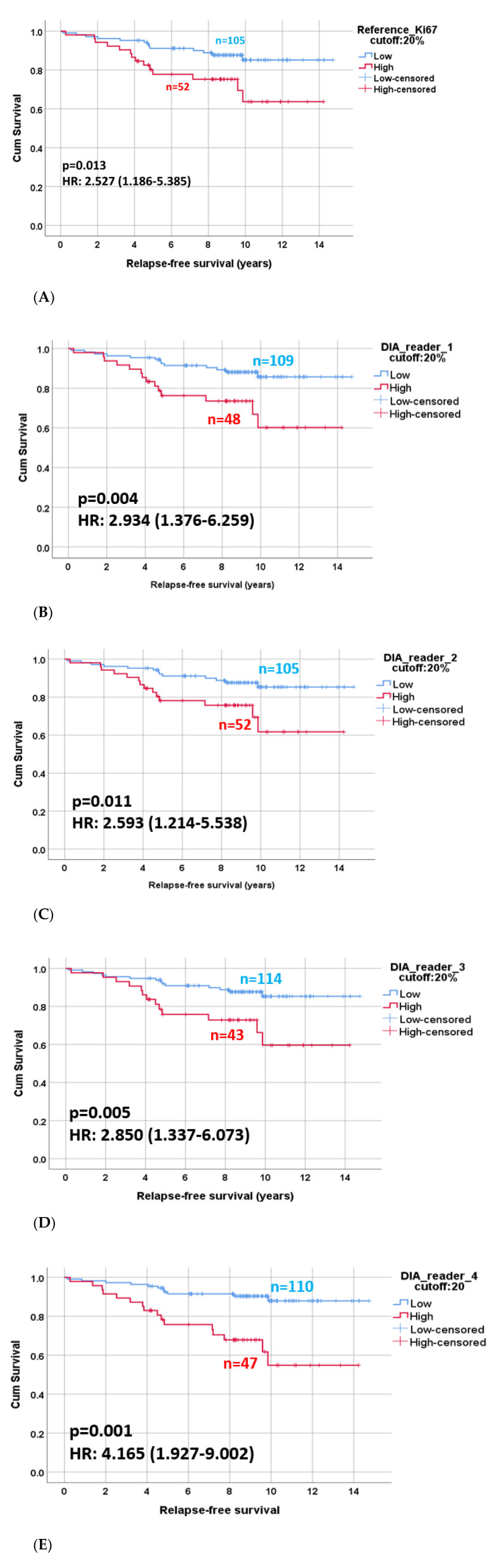
(**A**–**E**) Kaplan–Meier curves illustrating recurrence-free survival (RFS) based on Ki67 scoring divided as low or high according to the threshold of 20%. (**A**) Reference Ki67. (**B**–**E**) Ki67 scoring based on four different algorithms.

The univariable survival analysis supported by Kaplan–Meier curves showed significant differences in RFS among patient groups with high and low Ki67 scores for each DIA algorithm (*p* ≤ 0.011 for all comparisons). The number of patients grouped as Ki67 low and Ki67 high was very similar among the algorithms (Figure 4). The hazard ratio values of the four algorithms (2.593–4.165) overlapped with that of the reference scoring (2.527) (Figure 4). The Kaplan–Meier analysis for RFS with Ki67 scoring reached a statistical power of 0.80, which was considered powered enough. In order to further investigate the independent prognostic potential of DIA global scoring, we performed a multivariable Cox regression analysis (Table 2). Adjusting the regression model to tumor size (pT1, pT2, pT3), Nottingham histological grade (1, 2, 3) and lymph node status (pN0, pN1, pN2, pN3), all DIA Ki67 algorithms, including the reference Ki67 scoring, remained independent prognostic markers of RFS (*p* ≤ 0.018 for all comparisons) besides lymph node status (*p* ≤ 0.05).

## 4. Discussion

Many efforts have been made to implement Ki67 in the clinical management of breast cancer. Various studies have attempted to find an association between Ki67 expression and prognostic parameters, such as hormone receptor status, lymph node status, tumor size or patient age, and demonstrated controversial results [17,18,19]. Furthermore, dividing the Ki67 score into three categories was suggested as an alternative to mitotic count in a breast carcinoma histological grading system [18].

It is now acknowledged that Ki67 index is an important marker with prognostic and potential predictive value in breast cancer that differs depending on the therapeutic approach. It might also be an independent factor to predict pathological complete response [20]. According to the latest St. Gallen International Breast Cancer Conference, Ki67 should be included in routine pathology reports for ER-positive HER2-negative T1–2 N0–1 tumors with a more formal evaluation method referring to the IKWG’s recommendations [3,21].

However, Ki67 is a controversial biomarker in terms of evaluation. In the literature, many practical issues have emerged regarding Ki67 assessment, which limits its clinical implementation in breast cancer treatment decisions [22]. The most discussed factor that hinders the clinical usage of Ki67 is interobserver variability due to the varying scoring methods, selection of tumor areas and subjective assessment of staining positivity [23,24]. There are several DIA platforms offering a solution for scoring Ki67. Although studies have shown both high correlations between machine-read and manual scores and good interplatform agreement [25,26], none of these platforms have reached clinical utility yet. In our study, we confirmed the prognostic potential of the automated Ki67 scoring guideline proposed by the IKWG. We found similar results for four independent algorithms created by four observers. We also demonstrated that high reproducibility can be reached using QuPath in Ki67 analysis of breast cancer, similarly to the IKWG study implementing the same guideline in 17 different laboratories [8]. Global scoring with DIA may help to overcome the obstacle of low reproducibility, excluding the most subjective parts in the scoring process. For heterogenous tumors, concordance between the observers is generally lower than that for homogenous ones, especially for hotspot scoring with both eyeballing methods and DIA [27]. In an international study of 30 ER-positive breast cancer cases, different DIA platforms were chosen by different laboratories, yet they achieved high ICCs for global scores [28]. Although machine learning-based tools can aid scoring-related reproducibility issues, more focus has to be placed on pre-analytical and analytical processes to achieve complete standardization of Ki67 assessment [23].

There are several limitations to this work. Most significantly, this is a retrospective single-center study, and a multi-institutional study is needed to confirm the clinical validity of the applied guideline. Further studies are needed to investigate whether the used DIA instructions in this study can be implemented with refinements in pathology practice, especially focusing on lab-specific machine learning training requirements, lab-specific immunohistochemistry protocol differences or differences in the choice of slide scanners. Furthermore, the machine learning algorithm used in this study is susceptible to cell assignment error during cell classification.

In conclusion, we demonstrated that good reproducibility can be reached among pathologists using the IKWG automated Ki67 scoring guideline, achieving similar ICC values as in the study of the IKWG [8]. Moreover, we also showed the prognostic potential of the automated IKWG scoring guideline in an independent breast cancer cohort. The advantage of this method is that it is easily implemented with a freely accessible platform. Our study provides the first independent validation of the IKWG guideline with multiple observers. Finally, a general DIA standardization guideline for biomarker assessment and a prospective study to test the method´s clinical utility are fundamental.

## Figures and Tables

**Table 1:** Patient demographics and tumor characteristics.

	*n*	%
**Total cases**	157	100
**Patient median age (range)**	59 (28–79)	
**Tumor mean diameter (mm)**	25	
**Tumor median diameter (mm)**	22	
Histological grade *		
**1**	27	17
**2**	84	54
**3**	46	29
Tumor size **		
**pT1**	63	40
**pT2**	86	55
**pT3**	8	5
Lymph node status **		
**pN0**	87	55
**pN1**	51	32
**pN2**	15	10
**pN3**	4	3

* Nottingham histological score. ** Pathological tumor-node-metastasis (pTNM) classification according to 8th Edition of American Joint Committee on Cancer (AJCC) breast cancer staging system.

**Table 2:** Multivariable Cox regression analysis for clinical pathological factors and digitally scored Ki67.

Variables	*p*-Value	HR	95.0% CI for Exp(B)	
			**Lower**	**Upper**
HG 1	0.623			
HG 2	0.703	1.348	0.291	6.249
HG 3	0.829	0.816	0.128	5.191
Tumor size < 20 mm	0.675			
20–50 mm	0.434	1.448	0.573	3.655
>50 mm	0.466	1.859	0.351	9.838
LN without metastasis	0.021			
1–3 metastasis in LN	0.544	1.336	0.525	3.402
4–9 metastasis in LN	0.003	4.742	1.696	13.258
≥10 metastasis in LN	0.151	3.947	0.607	25.649
Reference Ki67	0.017	3.72	1.263	10.957
DIA reader 1	0.006	4.835	1.587	14.734
DIA reader 2	0.018	3.597	1.246	10.386
DIA reader 3	0.017	3.96	1.273	12.324
DIA reader 4	0.001	8.074	2.842	22.937

HG: histological grade; LN: lymph nodes; DIA: digital image analysis.

## Data Availability

The data is within the article available from the authors upon request but may require data transfer agreements. No personalized health information will be shared.

## Supplementary Materials

The following are available online at https://www.mdpi.com/article/10.3390/biom11111612/s1, Supplementary File 1: The list of features used in the random-trees-based classification.

## Abbreviations

IKWG	International Ki67 in Breast Cancer Working Group
DIA	Digital image analysis
ICC	Intraclass correlation coefficient
WS	Whole-slide section
HE	Hematoxylin and eosin
DAB	3,3’-diaminobenzidine
CI	Confidence interval
RFS	Relapse-free survival
pTNM	Pathological Tumor-Node-Metastasis
AJCC	American Joint Committee on Cancer
