# Supplementary material for: Independent Clinical Validation of the Automated Ki67 Scoring Guideline from the International Ki67 in Breast Cancer Working Group
Source: Biomolecules. 2021 Oct 30;11(11):1612. doi: 10.3390/biom11111612 (PMC8615770; doi:10.3390/biom11111612)
Supplement: Supplementary file 1 [file biomolecules-11-01612-s001.zip › biomolecules-1426602-supplementary.pdf]

Supplementary File 1

The list of features used in the random trees-based classification.

Nucleus: Area
Nucleus: Perimeter
Nucleus: Circularity
Nucleus: Max caliper
Nucleus: Min caliper
Nucleus: Eccentricity
Nucleus: Hematoxylin OD mean
Nucleus: Hematoxylin OD sum
Nucleus: Hematoxylin OD std dev
Nucleus: Hematoxylin OD max
Nucleus: Hematoxylin OD min
Nucleus: Hematoxylin OD range
Nucleus: DAB OD mean
Nucleus: DAB OD sum
Nucleus: DAB OD std dev
Nucleus: DAB OD max
Nucleus: DAB OD min
Nucleus: DAB OD range
Cell: Area
Cell: Perimeter
Cell: Circularity
Cell: Max caliper
Cell: Min caliper
Cell: Eccentricity
Cell: DAB OD mean
Cell: DAB OD std dev
Cell: DAB OD max
Cell: DAB OD min
Cytoplasm: DAB OD mean
Cytoplasm: DAB OD std dev
Cytoplasm: DAB OD max
Cytoplasm: DAB OD min
Nucleus/Cell area ratio

Smoothed: 25 µm: Nucleus: Area
Smoothed: 25 µm: Nucleus: Perimeter
Smoothed: 25 µm: Nucleus: Circularity
Smoothed: 25 µm: Nucleus: Max caliper
Smoothed: 25 µm: Nucleus: Min caliper
Smoothed: 25 µm: Nucleus: Eccentricity
Smoothed: 25 µm: Nucleus: Hematoxylin OD mean
Smoothed: 25 µm: Nucleus: Hematoxylin OD sum
Smoothed: 25 µm: Nucleus: Hematoxylin OD std dev
Smoothed: 25 µm: Nucleus: Hematoxylin OD max
Smoothed: 25 µm: Nucleus: Hematoxylin OD min
Smoothed: 25 µm: Nucleus: Hematoxylin OD range
Smoothed: 25 µm: Nucleus: DAB OD mean
Smoothed: 25 µm: Nucleus: DAB OD sum
Smoothed: 25 µm: Nucleus: DAB OD std dev
Smoothed: 25 µm: Nucleus: DAB OD max
Smoothed: 25 µm: Nucleus: DAB OD min
Smoothed: 25 µm: Nucleus: DAB OD range
Smoothed: 25 µm: Cell: Area
Smoothed: 25 µm: Cell: Perimeter
Smoothed: 25 µm: Cell: Circularity
Smoothed: 25 µm: Cell: Max caliper
Smoothed: 25 µm: Cell: Min caliper
Smoothed: 25 µm: Cell: Eccentricity
Smoothed: 25 µm: Cell: DAB OD mean
Smoothed: 25 µm: Cell: DAB OD std dev
Smoothed: 25 µm: Cell: DAB OD max
Smoothed: 25 µm: Cell: DAB OD min
Smoothed: 25 µm: Cytoplasm: DAB OD mean
Smoothed: 25 µm: Cytoplasm: DAB OD std dev
Smoothed: 25 µm: Cytoplasm: DAB OD max
Smoothed: 25 µm: Cytoplasm: DAB OD min
Smoothed: 25 µm: Nucleus/Cell area ratio
Smoothed: 25 µm: Nearby detection counts

Smoothed: 50 µm: Nucleus: Area
Smoothed: 50 µm: Nucleus: Perimeter
Smoothed: 50 µm: Nucleus: Circularity
Smoothed: 50 µm: Nucleus: Max caliper
Smoothed: 50 µm: Nucleus: Min caliper
Smoothed: 50 µm: Nucleus: Eccentricity
Smoothed: 50 µm: Nucleus: Hematoxylin OD mean
Smoothed: 50 µm: Nucleus: Hematoxylin OD sum
Smoothed: 50 µm: Nucleus: Hematoxylin OD std dev
Smoothed: 50 µm: Nucleus: Hematoxylin OD max
Smoothed: 50 µm: Nucleus: Hematoxylin OD min
Smoothed: 50 µm: Nucleus: Hematoxylin OD range
Smoothed: 50 µm: Nucleus: DAB OD mean
Smoothed: 50 µm: Nucleus: DAB OD sum
Smoothed: 50 µm: Nucleus: DAB OD std dev
Smoothed: 50 µm: Nucleus: DAB OD max
Smoothed: 50 µm: Nucleus: DAB OD min
Smoothed: 50 µm: Nucleus: DAB OD range
Smoothed: 50 µm: Cell: Area
Smoothed: 50 µm: Cell: Perimeter
Smoothed: 50 µm: Cell: Circularity
Smoothed: 50 µm: Cell: Max caliper
Smoothed: 50 µm: Cell: Min caliper
Smoothed: 50 µm: Cell: Eccentricity
Smoothed: 50 µm: Cell: DAB OD mean
Smoothed: 50 µm: Cell: DAB OD std dev
Smoothed: 50 µm: Cell: DAB OD max
Smoothed: 50 µm: Cell: DAB OD min
Smoothed: 50 µm: Cytoplasm: DAB OD mean
Smoothed: 50 µm: Cytoplasm: DAB OD std dev
Smoothed: 50 µm: Cytoplasm: DAB OD max
Smoothed: 50 µm: Cytoplasm: DAB OD min
Smoothed: 50 µm: Nucleus/Cell area ratio
Smoothed: 50 µm: Nearby detection counts
